# Health of the City of New Orleans

**Published:** 1847-12

**Authors:** 


### RECORD OF MEDICAL SCIENCE.

*Health of the City of New Orleans.*—We shall continue hereafter, as heretofore, to make a few passing remarks upon the sanitary condition of our city. In our September number, it was stated that the Board of Health had declared the yellow fever to be epidemic. Events which have transpired since that announcement, have fully verified, we regret to say, the assertion of the Board.

The fever made its appearance about the 1st of July, and began to decline, in accordance with the laws of epidemics, about the latter part of September; and by the 1st of October, the deaths daily were about ten. Thus the epidemic, as such, raged about six or seven weeks. It attacked many who had passed unaffected through the season of 1839 and '41; some who had been permanent residents for several years, fell victims to the disease. In some of its features it differed from former epidemics.

We had fewer cases of black vomit; and many who were attacked with this usually fatal symptom, recovered. In many cases, the fever terminated in 24 hours; in others it raged for 56 or 72 hours Nor was the issue of the case materially influenced by the duration of the fever. Since many in whom the fever continued for three days, recovered as promptly as those in whom it ceased at the end of 24 hours. Throughout the disease, the head-symptoms were striking and obstinate ; and many, very many, succumbed with all the symptoms of congestion of brain.

As it is not our object to go into a description of the disease, we shall confine ourselves to the statistics on the subject. After much labor and great care, we have compiled from the published reports of the Board of Health the following statement, which will speak for itself.

Interments in the city of New Orleans, from the 3rd of July, to the 18th October, 1847, inclusive.

| | | | | | |
|---|---|---|---|---|---|
| For the week ending | 10th July. | Total | 138, | 5 | of yellow fever, |
| " " " " | 17th " | " | 143, | 6 | " " |
| " " " " | 24th " | " | 131, | 16 | " " |
| " " " " | 31st " | " | 177, | 47 | " " |
| " " " " | 8th Aug. | " | 263, | 118 | " " |
| " " " " | 15th " | " | 353, | 197 | " " |
| " " " " | 22d " | " | 443, | 322 | " " |
| " " " " | 29th " | " | 461, | 328 | " " |
| " " " " | 5th Sept. | " | 540, | 435 | " " |
| " " " " | 12th " | " | 491, | 355 | " " |
| " " " " | 19th " | " | 257, | 169 | " " |
| " " " " | 26th " | " | 181, | 85 | " " |
| " " " " | 3rd Oct. | " | 149, | 61 | " " |
| " " " " | 10th " | " | 126, | 44 | " " |
| From the 10th to the | 18th " | " | 148, | 53 | " " |
| Total . . . . . . . . . . | | | 3990, | 2241 | of yellow fever. |

Interments in the city of Lafayette, from 26th of July to 21st of September, 1847, inclusive. Total, 793, of which 498 were of yellow fever. Thus making the total of deaths from all diseases during the time specified—in both cities—4,783, of which 2,739 were from yellow fever.

The above table will convey quite a correct idea of the state of health of our population from the 3d of July to the 18th of October, 1847.

From the foregoing table it will appear that the epidemic reached its acme about the 1st of September, and after that date it gradually declined. During the prevalence of the fever, it has been computed that between twenty and twenty-five thousand persons were attacked with the disease ; this, however, is more a matter of conjecture than accurate calculation.

On the 18th of October, the Board of Health published the following statement :

*Meeting of the Board of Health, October 18th, 1847.*

The Board of Health feels authorized to make the announcement that the yellow fever, which has been prevailing for some months as an *epidemic*, has, for some time, ceased to exhibit this character, and as such has now disappeared. At the same time it is proper to state that the *sporadic* cases, which have always been seen for one or two months after the disappearance of epidemic yellow fever, must still be expected to prevail.

Signed, W. Stone, *Chairman.*

W. T. Brent, *Secretary pro tem.*

As the yellow fever declines, our ordinary autumnal diseases begin to make their appearance. We have now under treatment two cases of typhoid fever in private practice; they are very obstinate, one 17 days standing, and not yet convalescent. Since the commencement of the fall season, the sky has been clear, the air cool and bracing, and but little rain to interrupt out-door business.—*New Orleans Medical and Surgical Journal.*

